# Regulated control of virus replication by 4-hydroxytamoxifen-induced splicing

**DOI:** 10.3389/fmicb.2023.1112580

**Published:** 2023-03-13

**Authors:** Zhenghao Zhao, Busen Wang, Shipo Wu, Zhe Zhang, Yi Chen, Jinlong Zhang, Yudong Wang, Danni Zhu, Yao Li, Jinghan Xu, Lihua Hou, Wei Chen

**Affiliations:** ^1^Beijing Institute of Biotechnology, Beijing, China; ^2^Qingdao Special Servicemen Recuperation Center of PLA Navy, Qingdao, Shandong, China

**Keywords:** vesicular stomatitis virus, intein, small molecule switch, virus regulation, post-translation regulation

## Abstract

Designing a modified virus that can be controlled to replicate will facilitate the study of pathogenic mechanisms of virus and virus–host interactions. Here, we report a universal switch element that enables precise control of virus replication after exposure to a small molecule. Inteins mediate a traceless protein splicing–ligation process, and we generate a series of modified vesicular stomatitis virus (VSV) with intein insertion into the nucleocapsid, phosphoprotein, or large RNA-dependent RNA polymerase of VSV. Two recombinant VSV, LC599 and LY1744, were screened for intein insertion in the large RNA-dependent RNA polymerase of VSV, and their replication was regulated in a dose-dependent manner with the small molecule 4-hydroxytamoxifen, which induces intein splicing to restore the VSV replication. Furthermore, in the presence of 4-hydroxytamoxifen, the intein-modified VSV LC599 replicated efficiently in an animal model like a prototype of VSV. Thus, we present a simple and highly adaptable tool for regulating virus replication.

## 1. Introduction

Naturally occurring viruses can be artificially modified for different purposes using a variety of methods. For example, a series of Adeno-associated viruses have been modified for a variety of gene therapies, and Adenoviruses have been modified to become replication-deficient, carrying antigens that can be used as vaccines (Tatsis and Ertl, 2004). Attenuated recombinant vesicular stomatitis virus (rVSV) can be generated by combining the deletion of the cytoplasmic tail of the G protein, gene translocation, and noncytopathic M gene mutations (Clarke and Nasar, 2007; Cooper and Wright, 2008). Live but replication-incompetent influenza A virus can be generated by applying genetic code expansion to the influenza virus genome, which maintains the reproductive potential of the progeny viruses in a special cell line but is replication-incompetent *in vivo* (Si and Xu, 2016). Those methods can attenuate viruses with varying degrees or totally remove the replication capacity of the viruses, and these modified viruses are different from prototype viruses in virulence and protein expression, especially *in vivo*.

We aim to develop a molecular switch that enables precise control of virus replication and manipulates the replication of viruses in an improved way, which can be achieved by intein splicing. Inteins are protein domains found in nature with the capability to carry out the process of protein splicing (Shah and Muir, 2014). Protein splicing is a post-translation biochemical modification that results in the cleavage and formation of peptide bonds between precursor polypeptide segments flanking the intein (Romero-Casanas and Gordo, 2020). Protein splicing excises the internal region of the precursor protein, which is then followed by the ligation of the N-extein and C-extein fragments, resulting in two polypeptides, the excised intein and the new polypeptide produced by joining the C-extein and N-extein (Perler and Davis, 1994). In many inteins, this biochemical reaction is spontaneous and not requiring any external factor or energy source to work (Myscofski and Dutton, 2001). Liu David’group previously evolved inteins that undergo protein splicing only in the presence of 4-hydroxytamoxifen (4-HT) (Buskirk and Ong, 2004). These inteins were developed by inserting the human estrogen receptor ligand-binding domain into the *Mycobacterium tuberculosis* RecA intein and evolving the resulting inactive fusion protein into a conditionally active intein that requires the presence of 4-HT. The modified intein has 413 amino acids, and its splicing leaves behind a single cysteine residue (Davis and Pattanayak, 2015). It is well confirmed that the 4-HT-dependent intein splicing is a post-translational process and rapidly regulates protein function in mammalian cells. However, the applications of this molecular switch focus on the protein level (Buskirk and Ong, 2004; Yuen and Rodda, 2006; Peck and Chen, 2011), and few attempts have been made to regulate the replication of the virus through this switch.

Vesicular stomatitis virus (VSV) belongs to the family Rhabdoviridae in the genus *Vesiculovirus*, and it is an enveloped, bullet-shaped virus with a negative, single-stranded, 11 kbp RNA genome encoding five structural proteins: nucleocapsid (N), phosphoprotein (P), matrix protein (M), glycoprotein (G), and large RNA-dependent RNA polymerase (L) (Rodriguez and Pauszek, 2002; Walker and Firth, 2015). Since VSV was recovered from a full-length cDNA clone (Lawson and Stillman, 1995; Whelan and Ball, 1995), it is well developed as a widely used research tool, as well as a vaccine platform (Garbutt and Liebscher, 2004; Jones and Feldmann, 2005; Mire and Geisbert, 2013; Case and Rothlauf, 2020; Yahalom-Ronen and Tamir, 2020) and oncolytic vector (Barber, 2005; Durham and Mulgrew, 2017). The genome of VSV is small and easy to manipulate (Haglund and Forman, 2000), and VSV replicates quickly to high titers in most mammalian cell lines (Liu and Cao, 2021). In this study, we used VSV as a model virus to explore whether 4-HT-dependent intein could regulate the replication of the virus *in vitro* and *in vivo*.

## 2. Methods

### 2.1. Cell culture

BHK-21 and Vero E6 cells were obtained from the ATCC. BHK-T7 cells were constructed by stably transfecting the T7 RNA polymerase gene into BHK-21 cells, and the detailed procedure is presented in the Supplementary materials. All cells were cultured at 37°C in a 5% CO_2_ incubator and maintained in Dulbecco’s modified Eagle’s medium (Thermo Scientific, United States) supplemented with 10% fetal bovine serum (Thermo Scientific, United States), penicillin (100 units/mL), and streptomycin (100 μg/mL). Furthermore, no mycoplasma was contained in all cell lines, which had been tested recently.

### 2.2. Plasmid construction

The sequences of 4-HT-dependent intein were synthesized (Genscript, CN) and amplified by PCR using the following primers: forward, 5′- TGCCTTGCCGAGGGTAC -3′ and reverse, 5′- GTTGTGCACGACAACCC -3′. The vector pMD-eGFP or pMD-luciferase was linearized by inverse PCR with 5′ homology region: 5′- GGGTTGTCGTGCACAAC -3′ and 3′ homology region: 5′- GTACCCTCGGCAAGGCA −3′ at different primer pairs for each intein insertion site. Homologous recombination of the linearized vector and intein fragment yielded intact plasmids pMD-eGFP-Intein and pMD-Luciferase-Intein. The recombinant VSV intein plasmids were constructed in the same way with a longer extension period (8 min for 72°C). All newly constructed plasmids were verified by DNA sequencing. The construction of recombinant VSV plasmids and support plasmids is described in Supplementary materials.

### 2.3. Recombinant VSV recovery

Recombinant VSV was recovered as described previously (Lawson and Stillman, 1995). In brief, BHK-T7 cells in a 6-well plate were infected with recombinant vaccinia virus vTF7-3 expressing T7 RNA polymerase. Each recombinant VSV plasmid (5 μg), together with support plasmids pBlu-N (3 μg), pBlu-P (5 μg), pBlu-G (4 μg), and pBlu-L (1 μg) containing T7 promoters, was transfected into vaccinia virus-infected cells, and the 4-HT was added to the transfection media to a final concentration of 10 μM. After 48 h, the supernatants were collected and filtered through a 0.2 μm pore diameter filter two times to remove vTF7-3, and it was defined as P1 generation virus and passaged onto fresh BHK-T7 cells for G complete VSV. As for delta-G viruses, Vero E6 cells in a 6-well plate were transfected with a plasmid (pCAG-G, 2 μg) that expresses VSV G protein for 12 h, then the delta-G viruses passaged onto the cells.

### 2.4. Microscopy and luciferase assay

Brightfield images and GFP images were captured on the Cytation 5 (Biotek, United States), according to the manufacturer’s specifications. As for the luciferase assay, taking the detection of 4-HT regulation capacity on the P1 generation virus as an example, cells were seeded at 2.5 × 10^5^ cells/well of a 24-well plate, cultured for 12–16 h, and grown to 95–100% confluence. The culture medium was removed, and 100 μL P1 virus plus 200 μL DMEM was added to the cells. After infection for 2 h, the supernatant was removed, cells were washed, and 500 μL of DMEM containing 5% FBS with or without 10 μM 4-HT was added to each well. In the second-round infection, cells were seeded at 5 × 10^4^ cells/well of a 96-well plate, cultured for 12–16 h, and grown to 95–100% confluence. Then 10-fold serial dilutions (from 10^1^ to 10^6^) of progeny viruses would be performed using culture media with or without 10 μM 4-HT. Moreover, 100 μL virus stocks of each dilution were added to infect BHK-T7 cells in the presence or absence of 4-HT. The status of the cell was observed before lysing, and the lowest dilution (10^1^) for which no broken cells were observed was selected. Cells of the selected dilution were lysed with a cell lysis buffer (Promega), and the lysates were transferred to a 96-well white plate. Luciferase activities were determined with a luciferase assay reagent (Promega) and a GloMax luminometer (Promega).

### 2.5. Flow cytometry

After being infected with VSV (dG)-GFP/VSV (dG)-GFP-intein for 24 h, cells were collected and washed successively with PBS and resuspended in PBS, and data were acquired on a FACSCanto Plus (BD Biosciences, United States). At least 200,000 events were collected for each sample, and the data were analyzed with FlowJo 10 software.

### 2.6. TCID_50_ assay

Vero E6 cells were seeded at 1×10^5^ cells/well of a 96-well plate, cultured for 12-16 h and grown to 95%-100% confluence. Cells were infected with 10-fold serial dilutions of the virus suspension (four replicates of each dilution were used), and incubated at 37°C for 72 h. Cell monolayers were observed microscopically for the presence of CPE. The TCID_50_ endpoint dilution was calculated by the Reed–Muench method (Reed and Muench, 1938).

### 2.7. Virus growth curve analysis

Virus replication kinetics was evaluated with a one-step growth curve. Confluent monolayers of BHK-T7 cells grown in a 12-well plate were inoculated with the virus at an MOI of 1 for 1 h, followed by washing and then incubation with Dulbecco’s modified Eagle’s medium supplemented with 5% fetal bovine serum at 37°C for 72 h. Culture medium from a different well of infected cells was collected at 12, 24, 48, and 72 h after infection and stored at −80°C for titration by TCID_50_ assay.

### 2.8. Animal experiments

Animal experiments were performed according to the guidelines of the Institutional Experimental Animal Welfare and Ethics Committee. BALB/c mice aged 42–56 days were purchased from SPF Biotechnology (Beijing, CN) and used for all experimental groups. 100 TCID_50_ of virus in 10 μL volume was injected intracerebrally into mice under anesthetic conditions (Mishra and Byrareddy, 2020). Mice in the 4-HT^+^ group and PBS group were administered (intraperitoneal) daily of 0.4 μg 4-HT/g weight of the animal. All mice from each group were sacrificed on day 5 post-inoculation, and their brains were harvested, homogenized, and titered by TCID_50_ assay or qPCR.

### 2.9. *In vivo* imaging

Noninvasive *in vivo* multispectral fluorescence imaging was performed using the *In vivo* imaging system (IVIS) Spectrum (PerkinElmer) with Living Image software (version 4.4). D-Luciferin Firefly (PerkinElmer, dissolved in PBS at a concentration of 15 mg/mL) was intraperitoneally injected into each mouse at a working dose of 150 mg/kg, and then mice were anesthetized using 3% isoflurane (RWD, CN) in O_2_ at a flow rate of 3–5 μL/min for 10 min before being transferred to the imaging chamber for *in vivo* imaging. Luminescence was measured for 5 min (Zhang and Zhao, 2022). All measurements were performed in bioluminescence mode. For the quantification of the total radiant efficiency, a region of interest (ROI) was drawn around the brain, and radiant efficiency was measured.

### 2.10. Quantitative reverse transcription-polymerase chain reaction

To detect the VSV N mRNA levels in the brain of mice infected with VSV, total RNA was isolated from tissue cells using TRIzol (Invitrogen, United States). The reverse transcription reactions were carried out by Hiscript III Reverse Transcriptase (Vazyme, CN), according to the manufacturer’s specifications. Quantitative real-time PCR was carried out according to the Taq Pro Universal SYBR qPCR Master Mix (Vazyme, CN), with 20 μL of a reaction mixture containing primers specific for the VSV N gene: (forward: 5′-GATAGTACCGGAGGATTGACGACTA-3′ and reverse: 5′-TCAAACCATCCGAGCCATTC-3′) or GAPDH RNA (forward: 5′- GGTTGTCTCCTGCGACTTCA −3′ and reverse: 5′- TGGTCCAGGGTTTCTTACTCC -3′). The results were calculated using the 2^−△△CT^ (two-delta delta CT) method according to the manufacturer’s specifications.

### 2.11. Hematoxylin and eosin staining

Hematoxylin and eosin (HE) staining was conducted according to routine protocols. In brief, after deparaffinization and rehydration, tissue sections were stained with hematoxylin solution for 5 min followed by 5 dips in 1% acid ethanol (1% HCl in 75% ethanol) and then rinsed in distilled water. Then, the sections were stained with eosin solution for 3 min and followed by dehydration with graded alcohol and clearing in xylene. The mounted slides were then examined and photographed using a DS-FI2 (NIKON, JP).

### 2.12. Statistical analysis

Statistical analysis and graphs were made by GraphPad Prism8 software. The significance of the results was evaluated by the Mann–Whitney test between the two groups. A value of *p* of less than 0.05 was considered significant. *p < 0.05; **p < 0.01; ***p < 0.001; n.s., not significant. All results are expressed as means ± SDs of the means. Error bars indicated *N* > 2.

## 3. Results

### 3.1. Screening effective intein insertion sites on VSV

**Figure 1:**
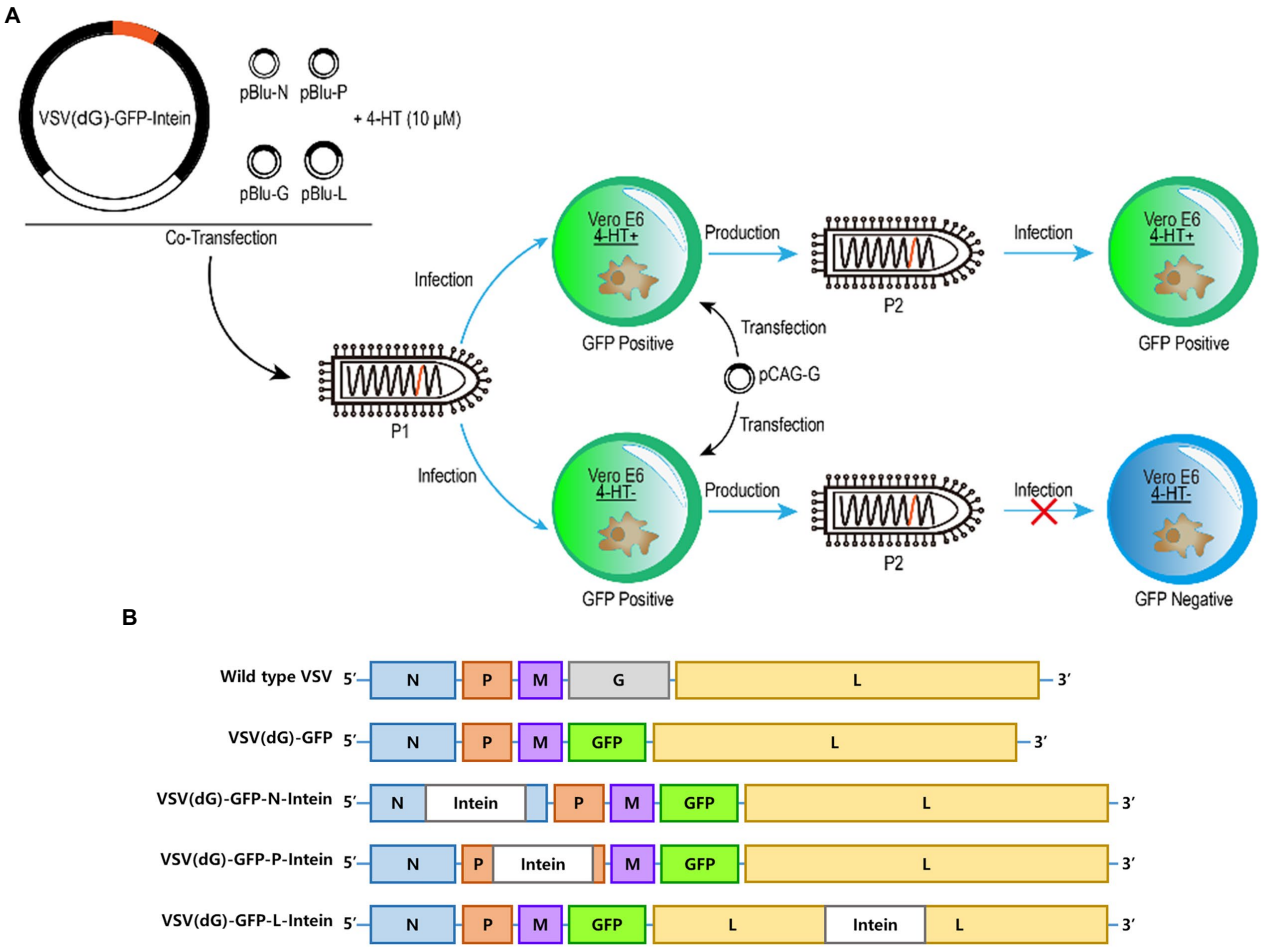
Experimental design and plasmids construction. **(A)** Schematic representation of the generation of recombinant 4-HT-regulated VSV that are characterized by high reproduction in 4-HT^+^ media but replication incompetence in 4-HT^−^ media. Moreover, the replication capacity was assayed by measuring the GFP expression of VSV (P2). **(B)** Virus genome schemes of VSV: wild type VSV, VSV (dG)-GFP, VSV (dG)-GFP-N-intein, VSV (dG)-GFP-P-intein, and VSV (dG)-GFP-L-intein.

First, we wanted to probe whether 4-HT-dependent intein could act as a molecular switch to moderate the replication of the VSV. The intein gene was inserted into different sites on VSV (dG)-GFP to construct recombinant VSV plasmids, which were cotransfected with other four support plasmids to package recombinant VSV (Passage 1, P1). Then, the Vero E6 cells transfected with VSV G protein expression plasmids were infected by the recombinant VSV virus (P1) in 4-HT^+^/4-HT^−^ media. The supernatant was collected to reinfect the Vero E6 cells. In addition, the difference in the GFP expression level between the 4-HT^+^ and 4-HT^−^ groups was measured to assay the replication capacity (Figure 1A).

The VSV genome which is present in the virion core is tightly encapsidated by the nucleocapsid protein (N) to form the ribonucleoprotein complex (Blumberg and Leppert, 1981). Moreover, the viral ribonucleoprotein serves as the template for transcription and replication by the viral RNA-dependent RNA polymerase, which is a complex of the large polymerase protein (L) and phosphoprotein (P) (Das and Pattnaik, 2005). Therefore, we selected N, P, and L proteins as the target-protein to insert intein. The recombinant VSV plasmids containing the intein gene were constructed based on the VSV (dG)-GFP (Figure 1B).

### 3.2. Two intein insertion sites in VSV L protein regulating the replication of VSV

**Figure 2:**
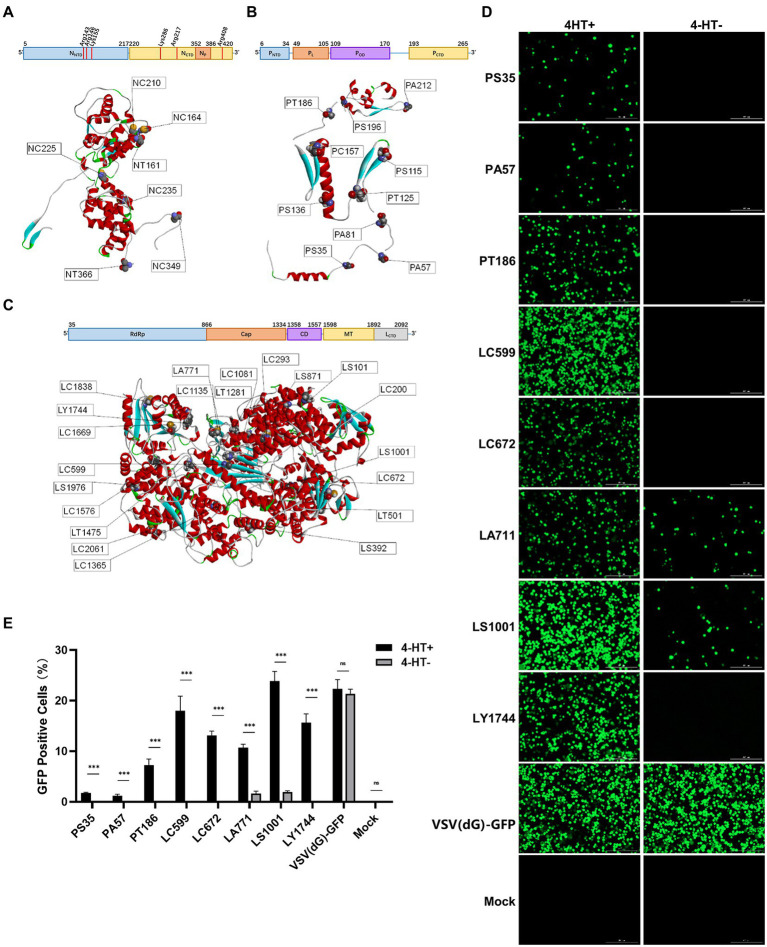
Screening regulation of intein insertion sites in viral proteins of VSV. **(A–C)** The structures and function domains of VSV nucleoprotein **(A)**, phosphoprotein **(B)**, the large RNA polymerase **(C)**, and the intein insertion sites selected on the structures. N protein domains (Green and Zhang, 2006): N-terminal domain (N_NTD_); C-terminal domain (N_CTD_); and RNA-binding sites are shown in red vertical lines. P protein domains (Jenni and Bloyet, 2020): N-terminal domain (P_NTD_); L protein-binding domain (P_L_); oligomerization (P_OD_); and C-terminal domain (P_CTD_). L protein domains (Jenni and Bloyet, 2020): RNA-dependent RNA polymerase domain (RdRp); capping domain (Cap); connector domain (CD); methyltransferase (MT); and C-terminal domain (CTD). **(D)** The GFP expression level of the recombinant 4-HT-regulated VSV and the percentages of GFP-positive cells were analyzed by FACS **(E)**. Significance of results were evaluated by Mann Whitney test between two groups. *p* value less than 0.05 was considered significant. *p < 0.05; **p < 0.01; ***p < 0.001; n.s., not significant.

We selected 7/10/21 intein insertion sites across the structure of these three proteins (Figures 2A–C). The 38 recombinant VSV plasmids were used for VSV packaging, and 18 recombinant VSV plasmids could be rescued from the plasmids with three sites on N protein, six sites on P protein, and nine sites on L protein (Supplementary Figure S1).

Next, we tested the 4-HT regulating activity on these 18 recombinant VSV plasmids. More than half (10) of the rescued viruses could not generate progeny virions, no matter with or without 4-HT. The recombinant VSV with intein insertion site LC599/LS1001/LY1744 had the strongest replication capacity, and their progeny virions expressed the strongest green fluorescence when infected with Vero E6 cells (Figure 2D). The LS1001 site, however, escaped from 4-HT regulation to a certain degree, resulting in partly restored replication competence under 4-HT^−^ conditions (Figure 2E). The Vero E6 cells were infected by VSV (P2) for 36 h, and the proportion of GFP-positive cells was measured by flow cytometry. The results showed good conformity between the fluorescence and the flow cytometric analysis, with the best regulation activity on LC599 and LY1744 sites.

Comprehensively, considering the packaging efficacy, regulation activity, and replication capacity, we chose the intein insertion sites LC599 and LY1744 to explore the characteristic of recombinant 4-HT-regulated VSV.

### 3.3. *In vitro* replication regulation of the intern-modified VSV *via* 4-HT

**Figure 3:**
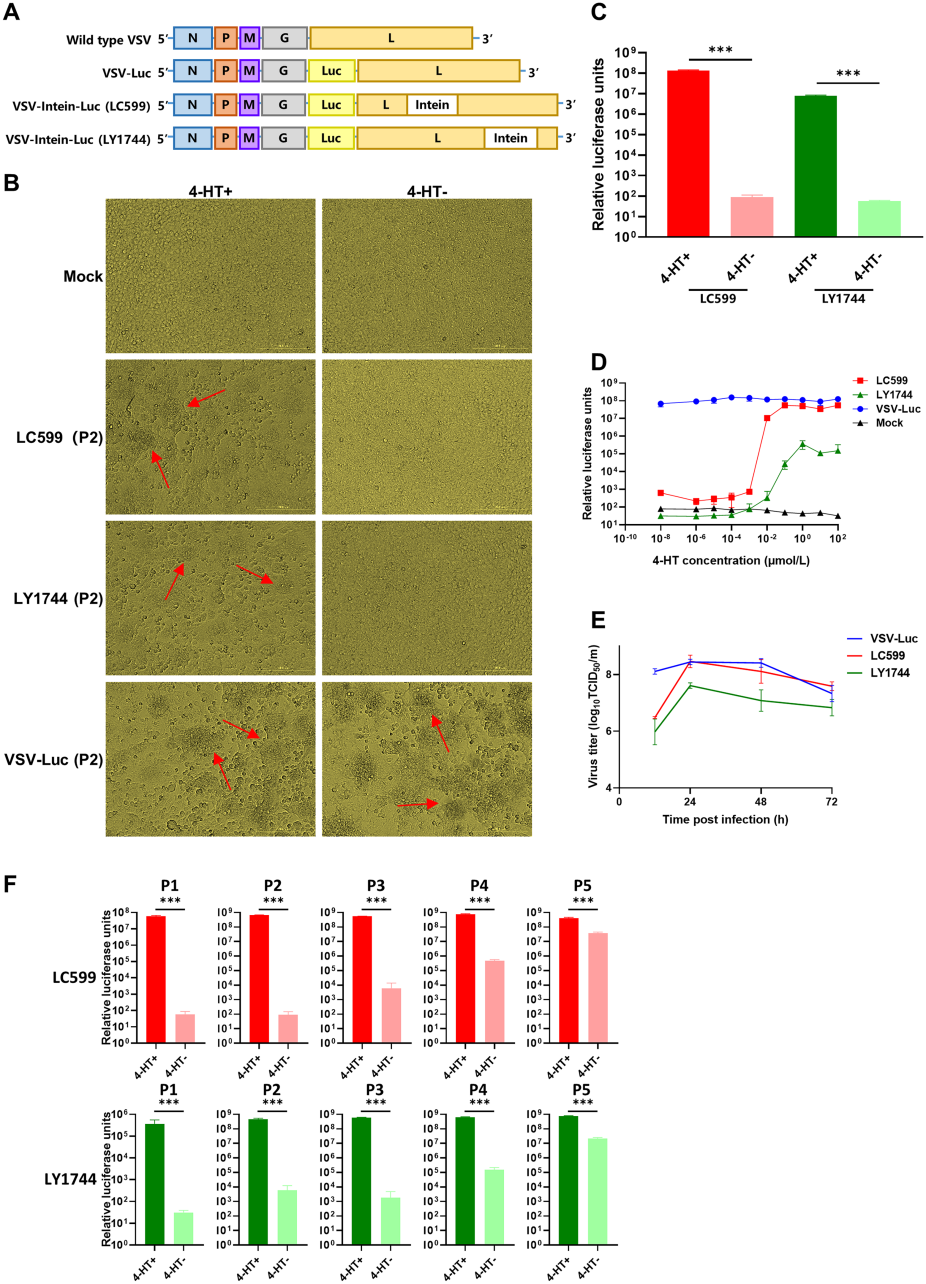
Characterization of the replication of the intein-inserted VSV in cells. **(A)** Virus genome schemes of VSVs: wild type VSV, VSV-Luc, LC599, and LY1744. **(B)** Cell morphology of BHK-T7 cells infected by LC599 (P2) and LY1744 (P2). The 4-HT^+^ group could form syncytia mediated by the expression of VSV G protein (red arrow). **(C)** 4-HT regulated the replication of LC599 (P2) and LY1744 (P2) in a dose-dependent manner. **(D)** The replication of LC599 (P2) and LY1744 (P2) with or without 4-HT. **(E)** The growth curve of VSV-Luc, LC599, and LY1744. **(F)** BHK-T7 cells were infected with P1 generation recombinant virus (4-HT^+^), and the culture supernatant harvested post-infection was defined as P2 generation. The culture supernatant harvested post-infection with P2 generation (4-HT^+^) was defined as P3 generation and so on. The 4-HT-regulated capacity on different passages of LC599 and LY1744 was detected. Significance of results were evaluated by Mann Whitney test between two groups. *p* value less than 0.05 was considered significant. *p < 0.05; **p < 0.01; ***p < 0.001; n.s., not significant.

We examined the characteristics of 4-HT-regulated VSV replication *in vitro*. We inserted the intein gene into the genome of VSV-Luc at the selected sites LC599 and LY1744, respectively (Figure 3A), and rescued the 4-HT-regulated VSV to passage on BHK-T7 cells. From the perspective of BHK-T7 cell morphology, we observed a significant formation of syncytia in cells infected by culture supernatants of LC599 (4-HT^+^) and LY1744 (4-HT^+^) (Figure 3B). Since the formation of syncytia was mediated by the expression of VSV G protein, it meant that the 4-HT-regulated VSV LC599 and LY1744 were replication-competent in the 4-HT^+^ media. While under the 4-HT^−^ conditions, LC599 (P2) and LY1744 (P2) could not express G protein (Figure 3B). As for VSV-Luc, it could infect BHK-T7 and form syncytia with or without 4-HT. As expected, the expression level of the exogenous gene luciferase was the same as the G protein and only expressed in the 4-HT^+^ groups in the secondary infection (Figure 3C).

We next explored the effect of different concentrations of 4-HT on virus replication. BHK-T7 cells were infected by LC599 and LY1744, with 4-HT at a series of concentrations, and the luciferase expression of LC599 (P2) and LY1744 (P2) was detected. The replication of both LC599 (P2) and LY1744 (P2) was 4-HT dose-dependent (Figure 3D). Two recombinant VSVs could replicate normally with a high concentration of 4-HT, but their replication capacity reduced as the concentration of 4-HT decreased, and finally totally lost. LC599 and LY1744 reached the highest replication capacity when the 4-HT concentration was above 0.1 and 1 μM relatively.

BHK-T7 cells were inoculated with virus VSV-Luc/LC599/LY1744 at an MOI of 1, and the culture medium from different times post-infection were collected to calculate and plot the growth curve (Figure 3E). At 12 h post-infection, progeny viruses could be detected in all three viruses, and the titer of VSV-Luc was higher than that of LC599 and LY1744, at this time point. The process of the intein splicing caused the delayed functioning of L protein and that may be the reason for the differences. Moreover, the peak virus titers were achieved at 24 h post-infection, which was approximately 3.2 × 10^8^ TCID_50_/mL for VSV-Luc and LC599 and 3.2 × 10^7^ TCID_50_/mL for LY1744. The peak titer of LY1744 was lower than that of VSV-Luc and LC599, which showed that the insertion of intein weakened its replication ability. After 48 h, the virus titers decreased as the cytopathic effect aggravated.

The regulation capacity of 4-HT on the LC599/LY1744 during serial passaging in cultured cells was detected (Figure 3F). LC599 and LY1744 were well regulated for at least five generations, and the progeny virus could not be detected in the culture supernatant for cells infected with the first or second passage of LC599/LY1744. The titers of the progeny virus of the fifth passage of LC599/LY1744 cultured with the 4-HT were 11.4-fold/59.4-fold higher than that of the titers of the progeny virus cultured without the 4-HT (Supplementary Figure S3).

### 3.4. *In vivo* replication regulation of the intern-modified VSV *via* 4-HT

**Figure 4:**
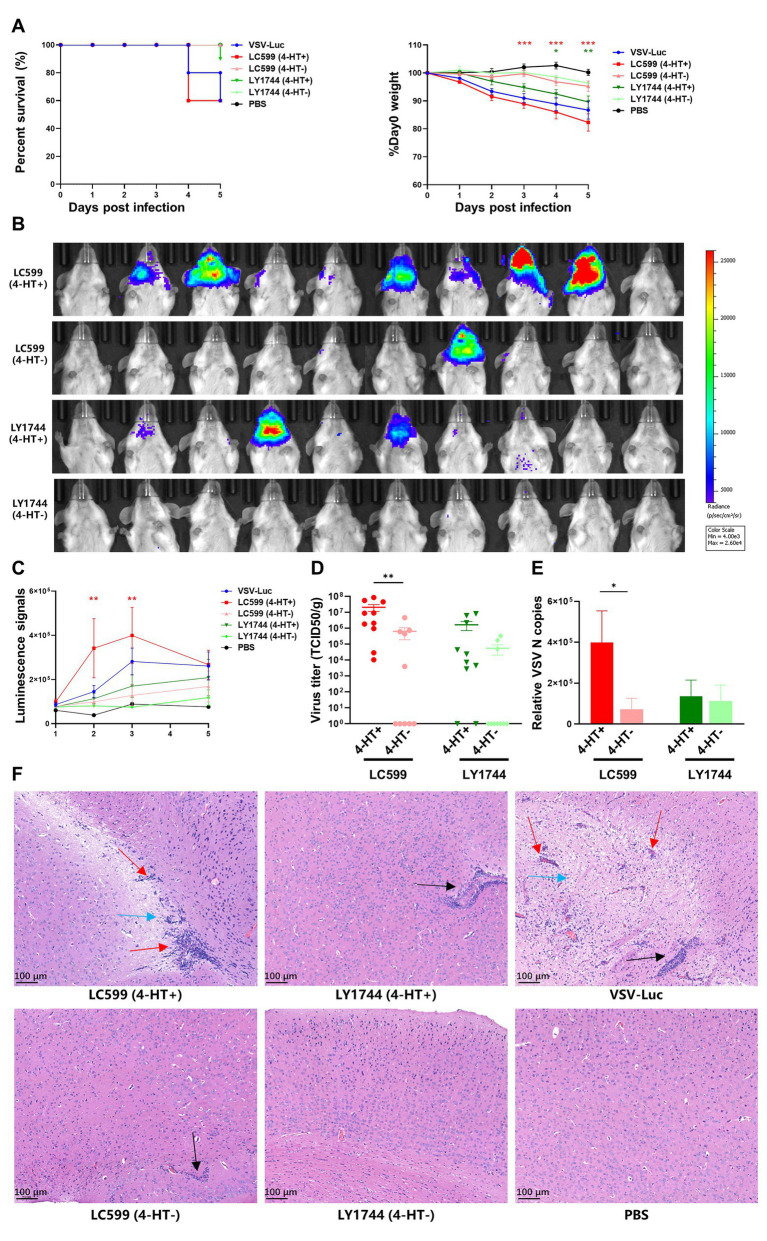
Characterization of the replication of the intern-modified VSV in mice. **(A)** Effect of intracranial (i.c.) virus infection with the indicated viruses on the survival rates and body weights of BALB/c mice (*n* = 10). **(B)** Luminescence imaging in challenged mice on day 3. Luminescence intensity (photons/s/cm2/sr) was represented in false colors. **(C)** Changes of the whole-brain luminescence signals. **(D)** Detection of the virus titers in the brain tissues of a mouse (*n* = 10) on day 5. **(E)** Viral RNA transcripts of N genes in the brain (5 dpi) were measured by quantitative real-time PCR. Data were plotted for individual mice (*n* = 10). **(F)** Representative brain sections were isolated on day 5 after the challenge of mice. Brain sections were stained with hematoxylin and eosin (HE), original magnification 10×. Lymphocyte infiltration (blue arrow), perivascular cuffing (red arrow), and lymphocyte aggregation (blue arrow). Significance of results were evaluated by Mann Whitney test between two groups. *p* value less than 0.05 was considered significant. *p < 0.05; **p < 0.01; ***p < 0.001; n.s., not significant.

Finally, we tested the *in vivo* regulation of the 4-HT-dependent VSV. Since intranasal (i.n.) infection of mice with VSV triggers the production of type I interferon in the Olfactory Bulb, which would constrain intracerebral virus to replicate and spread (Detje and Lienenklaus, 2015), we challenged the adult BALB/c mice intracerebrally with a dose of 100 TCID_50_. Infected mice were monitored for body weight changes and development of classic neurological symptoms, such as convulsion and limb paralysis, and death kinetics was plotted using the Kaplan–Meier survival curve (Figure 4A). The mice injected with VSV-Luc showed a significant decrease in body weight, and the mortality rate was 40% at day 5 post-challenge. The trend of body weight changes in the LC599 (4-HT^+^) group was similar to that of the VSV-Luc group, and these two groups had the same mortality rate. Mice infected with LY1744 (4-HT^+^) also showed a decrease in body weight, but the decrease was less significant than in the other two groups. The body weight of the LC599 (4-HT^−^) and LY1744 (4-HT^−^) groups decreased slightly and no mice died until day 5 post-infection. The luciferase expression in the brain could be detected in 7 out of 10 mice in the LC599 (4-HT^+^) group and 3 out of 10 mice in the LY1744 (4-HT^+^) group by live imaging at day 3 post-infection (Figure 4B), and only one mouse in the LC599 (4-HT^−^) group had detectable luciferase expression and none of the mice in LY1744 (4-HT^−^) group could detect luciferase expression. Then, the total luminescence intensity in the brain was calculated (Figure 4C; Supplementary Figure S4), which in the LC599 (4-HT^+^) group was significantly higher (*p* < 0.01) than the corresponding 4-HT^−^ group for day 2 and day 3 post-infection. At day 5, the expression of luciferase in the LC599 (4-HT^+^) group decreased, which may be due to the brain lesions caused by VSV infection.

The mice were sacrificed on day 5, and virus titers in the brain tissue were measured (Figure 4D). The virus could be detected in each mouse of the LC599 (4-HT^+^) group, and the titer was significantly higher than that of the LC599 (4-HT^−^) group, in which only half of the mice could detect the virus. Meanwhile, the relative expression level of VSV N protein mRNA was quantified (Figure 4E), and the virus gene copies of the LC599 (4-HT^+^) group were also higher than those of the LC599 (4-HT^−^) group, as expected. However, there were no significant differences between the LY1744 (4-HT^+^) and LY1744 (4-HT^−^) groups, both in the virus titers and the gene copies.

The results of the brain section showed the same trend (Figure 4F). In the LC599 (4-HT^+^) and VSV-Luc groups, the pathological response caused by VSV infection could be observed, including lymphocyte infiltration, perivascular cuffing, and lymphocyte aggregation. In the LC599 (4-HT^−^) and LY1744 (4-HT^+^) groups, only a small amount of lymphocyte aggregation was observed, and no obvious pathological changes in tissues could be detected. The section results of the LY1744 (4-HT^−^) group had no difference from the control group.

## 4. Discussion

Substantial improvements have been made toward the modification of virus replication, and normally the replication capacity of the virus is completely removed for the viral vectors. The development of an efficient switch that could regulate the viral replication will broaden the application of virus vector in gene therapy or vaccine experiments. We utilized 4-HT-dependent intein splicing to regulate the replication of VSV and constructed a recombinant 4-HT-regulated VSV LC599 that can be regulated by 4-HT in a post-translational way both *in vitro* and *in vivo*.

First, we screened the intein insertion sites on the VSV, and we found that the selection of intein insertion sites was a key factor affecting the efficiency of 4-HT regulation. The insertion of intein at different sites would lead to differences in the packaging efficiency of VSV, and the fluorescence intensity of cells infected by VSV (P1) with intein insertion sites on the L protein was higher than that of sites on the N or P protein. We compared the GFP expression level of the progeny viruses produced with or without 4-HT, and the two sites, LC599 and LY1744, had the best regulation activity, both were located on the L protein. While VSVs with intein inserted in the N protein could not produce progeny viruses.

The difference in the regulation activity of intein between VSV proteins may be due to the characteristics of VSV gene expression. VSV relies on RdRp for gene transcription. When polymerase moves along with RNA during VSV genome transcription, its efficiency decreases by 20–30% after each gene is transcribed (Iverson and Rose, 1981). Inserting intein into N or P protein, on the one hand, will reduce the expression of the downstream GFP gene. On the other hand, the unspliced intein may hinder the normal function of N or P protein. The N protein is the most abundant protein in VSV particles and plays an impotent role in the transcription and replication of VSV (Rainsford and Harouaka, 2010). Therefore, the possible incorrect structure or decreased expression level of N protein caused by inserting and incomplete splicing of intein greatly affects the replication of VSV. As for the insertion sites on the L protein, they have little impact on the expression of upstream genes. In addition to that, the expression level of L protein is excessive for VSV replication (Schnell and Buonocore, 1996), and the partial loss of its expression has little impact on virus replication.

We, then, explored the *in vitro* replication regulation of the intern-modified VSV LC599 and LY1744, and 4-HT showed strong regulation on both viruses. The replication ability and protein expression level of LC599 were largely restored with the existence of 4-HT, while they were significantly decreased without 4-HT. The result is different from the regulation on the protein level. This may be because the L protein of VSV is an RNA polymerase protein as well as a structural protein of VSV. LC599 is located in the polymerase functional area of the L protein, while LY1744 is located in the methyltransferase functional area of the L protein (Jenni and Bloyet, 2020). Inserting intein at these two sites will inactivate the corresponding enzyme function of the L protein. Moreover, we suppose that in addition to damaging the enzyme function, inserting intein on L protein will also hinder the binding between P protein and L protein. These two factors lead to the high regulation efficiency of small molecules on VSV.

In our experiment, the expression of foreign genes GFP/Luciferase of the P1 generation virus could be detected whether 4-HT was added or not (data not shown), which may be caused by the residual 4-HT in the P1 generation virus culture medium. The concentration of residual 4-HT in the culture media of cells infected with the P1 generation virus was lower than 100 nM. After the second-round infection, the residual 4-HT concentration in the progeny virus culture medium would be lower than 1 nM, which will not affect the gene expression of the virus (Figure 3D). The intein-inserted VSVs were passaged under the same conditions except for the addition of 4-HT. Compared with the 4-HT^+^ group, intein-inserted VSVs could not be passaged continuously in BHK-T7 cells without the addition of 4-HT, which fully confirmed the regulation capacity of 4-HT on the replication of recombinant intein-inserted VSVs.

We explored whether the regulation of 4-HT on LC599 and LY1744 was still effective *in vivo*. After intracerebrally injected in mice, LC599 (4-HT^+^) and VSV-Luc had the same lethality and similar pathogenicity, while LC599 (4-HT^−^) demonstrated only slight toxicity and was totally non-fatal. As expected, this was consistent with the regulatory characteristics of 4-HT on LC599 observed *in vitro*. The expression of luciferase by VSV in the brain of a mouse was monitored by *in vivo* imaging. On the first day after infection, the expression of luciferase in the neck was observed in both the 4-HT^+^ and 4-HT^−^ groups, and it was cleared after LC599 (4-HT^+^) could replicate in the brain of mice, and the luciferase expression was observed in the brain of seven mice on the third day after immunization. While LC599 (4-HT^−^) had a low replication capacity, only one mouse could observe luminescence at the same time point. The replication regulation of 4-HT on LC599 *in vivo* was further verified by the assessment of brain pathological changes of pathological sections.

However, all of the experiments showed that the replication level of LY1744 *in vivo* was low, regardless of whether 4-HT was added. We confirmed that the replication ability of LY1744 was slightly lower than that of the VSV-Luc virus at the cellular level. However, due to the influence of the immune system of mice and other factors, the gap in the replication ability was more significant *in vivo*. The replication ability of LY1744 had no significant difference between the 4-HT^+^ and 4-HT^−^ groups.

One of the mice infected with the LC599 virus in the absence of 4-HT expressed a significant level of luciferase in the brain on day 3 (Figure 4B), and we detected the 4-HT regulation capacity of the virus extracted from the brain tissue of the seventh mouse in the LC599 (4-HT^−^) group. We found that 4-HT still could regulate the replication of the virus. The expression level of luciferase in the offspring of the 4-HT^+^ group was 2.4 times higher than that of the 4-HT^−^ group (Supplementary Figure S5), though the 4-HT^−^ group was not totally replication-incompetent. The gene of VSV was extracted for RT-PCR, and the intein gene was not deleted from the L gene. Therefore, we speculated that the diminished regulatory capacity of intein was caused by adaptive point mutations in the VSV genome. The data at the cellular level also showed that the regulation capacity of 4-HT on LC599 would be reduced during serial passage. The replication of VSV is error-prone because RNA replicase base misincorporations are proofread very inefficiently (Steinhauer and Domingo, 1992). During passaging, uncontrollable viruses with low sensitivity to 4-HT would generate during replication. The high regulation capacity can be kept for at least five passages, while the security risks would be reduced but not completely eliminated for the *in vivo* experiment. The applications of the intein to regulate other viruses require the assessment of stability and safety.

There are several directions to take our work further. First, there are many sites on a virus that can regulate the replication and the workload of screening appropriate sites through the experiment is heavy, and it is likely to miss highly active sites. This process can be optimized by simulating the structure of the target protein inserted with intein through bioinformatics. Second, the mechanism of how small molecules regulates the replication of VSV deserves further exploration, which plays a more important role in the recovery of the structure or the recovery of the function.

Taken together, we applied a small molecule–dependent intein splicing technology to the regulation of viruses and constructed a recombinant 4-HT-regulated VSV LC599. LC599 is replication-competent in the presence of 4-HT and has a similar replication capacity, protein expression level, and pathogenicity to the VSV-Luc in the 4-HT^+^ media, while its replication capacity is significantly decreased in the absence of 4-HT. The regulation is highly efficient and dose-dependent in a posttranscriptional way, which can improve the safety of virus application without affecting the expression of the protein. Furthermore, it may become a general way for generating small molecular–regulated virus vaccines that can be used for almost any virus.

## Data availability statement

The original contributions presented in the study are included in the article/Supplementary material, further inquiries can be directed to the corresponding authors.

## Ethics statement

The animal study was reviewed and approved by the Animal Care and Use Committee of the Beijing Institute of Biotechnology.

## Author contributions

WC and LH conceived this study. ZHZ and BW designed the research protocol, wrote the manuscript, and performed the data acquisition and analysis. SW, ZZ, YC, JZ, YW, DZ, YL, and JX performed laboratory work. All authors read and approved the final version of the manuscript.

## Funding

This study was funded by grants from the Ministry of Science and Technology of the People’s Republic of China (No. 2018YFA0900804).

## Conflict of interest

The authors declare that the research was conducted in the absence of any commercial or financial relationships that could be construed as a potential conflict of interest.

## Publisher’s note

All claims expressed in this article are solely those of the authors and do not necessarily represent those of their affiliated organizations, or those of the publisher, the editors and the reviewers. Any product that may be evaluated in this article, or claim that may be made by its manufacturer, is not guaranteed or endorsed by the publisher.

## References

[ref1] Barber G. N. (2005). VSV-tumor selective replication and protein translation. Oncogene 24, 7710–7719. doi: 10.1038/sj.onc.1209042, PMID: 16299531

[ref2] Blumberg B. M. Leppert M. (1981). Interaction of VSV leader RNA and nucleocapsid protein may control VSV genome replication. Cells 23, 837–845. doi: 10.1016/0092-8674(81)90448-7, PMID: 6261959

[ref3] Buskirk A. R. Ong Y. C. (2004). Directed evolution of ligand dependence: small-molecule-activated protein splicing. Proc. Natl. Acad. Sci. U. S. A. 101, 10505–10510. doi: 10.1073/pnas.0402762101, PMID: 15247421 PMC489967

[ref4] Case J. B. Rothlauf P. W. (2020). Replication-competent vesicular stomatitis virus vaccine vector protects against SARS-CoV-2-mediated pathogenesis in mice. Cell Host Microbe 28, 465–474.e4. doi: 10.1016/j.chom.2020.07.018 32798445 PMC7391951

[ref5] Clarke D. K. Nasar F. (2007). Synergistic attenuation of vesicular stomatitis virus by combination of specific G gene truncations and N gene translocations. J. Virol. 81, 2056–2064. doi: 10.1128/JVI.01911-06, PMID: 17151112 PMC1797571

[ref6] Cooper D. Wright K. J. (2008). Attenuation of recombinant vesicular stomatitis virus-human immunodeficiency virus type 1 vaccine vectors by gene translocations and g gene truncation reduces neurovirulence and enhances immunogenicity in mice. J. Virol. 82, 207–219. doi: 10.1128/JVI.01515-07, PMID: 17942549 PMC2224363

[ref7] Das S. C. Pattnaik A. K. (2005). Role of the hypervariable hinge region of phosphoprotein P of vesicular stomatitis virus in viral RNA synthesis and assembly of infectious virus particles. J. Virol. 79, 8101–8112. doi: 10.1128/JVI.79.13.8101-8112.2005, PMID: 15956555 PMC1143711

[ref8] Davis K. M. Pattanayak V. (2015). Small molecule-triggered Cas9 protein with improved genome-editing specificity. Nat. Chem. Biol. 11, 316–318. doi: 10.1038/nchembio.1793, PMID: 25848930 PMC4402137

[ref9] Detje C. N. Lienenklaus S. (2015). Upon intranasal vesicular stomatitis virus infection, astrocytes in the olfactory bulb are important interferon Beta producers that protect from lethal encephalitis. J. Virol. 89, 2731–2738. doi: 10.1128/JVI.02044-14, PMID: 25540366 PMC4325722

[ref10] Durham N. M. Mulgrew K. (2017). Oncolytic VSV primes differential responses to immuno-oncology therapy. Mol. Ther. 25, 1917–1932. doi: 10.1016/j.ymthe.2017.05.006, PMID: 28578991 PMC5542805

[ref11] Garbutt M. Liebscher R. (2004). Properties of replication-competent vesicular stomatitis virus vectors expressing glycoproteins of filoviruses and arenaviruses. J. Virol. 78, 5458–5465. doi: 10.1128/JVI.78.10.5458-5465.2004, PMID: 15113924 PMC400370

[ref12] Green T. J. Zhang X. (2006). Structure of the vesicular stomatitis virus nucleoprotein-RNA complex. Science 313, 357–360. doi: 10.1126/science.1126953, PMID: 16778022

[ref13] Haglund K. Forman J. (2000). Expression of human immunodeficiency virus type 1 Gag protein precursor and envelope proteins from a vesicular stomatitis virus recombinant: high-level production of virus-like particles containing HIV envelope. Virology 268, 112–121. doi: 10.1006/viro.1999.0120, PMID: 10683333

[ref14] Iverson L. E. Rose J. K. (1981). Localized attenuation and discontinuous synthesis during vesicular stomatitis virus transcription. Cells 23, 477–484. doi: 10.1016/0092-8674(81)90143-4, PMID: 6258804

[ref15] Jenni S. Bloyet L. M. (2020). Structure of the vesicular stomatitis virus L protein in complex with its phosphoprotein cofactor. Cell Rep. 30, 53–60.e5. doi: 10.1016/j.celrep.2019.12.024 31914397 PMC7049099

[ref16] Jones S. M. Feldmann H. (2005). Live attenuated recombinant vaccine protects nonhuman primates against Ebola and Marburg viruses. Nat. Med. 11, 786–790. doi: 10.1038/nm1258, PMID: 15937495

[ref17] Lawson N. D. Stillman E. A. (1995). Recombinant vesicular stomatitis viruses from DNA. Proc. Natl. Acad. Sci. U. S. A. 92, 4477–4481. doi: 10.1073/pnas.92.10.4477, PMID: 7753828 PMC41967

[ref18] Liu G. Cao W. (2021). Vesicular stomatitis virus: From agricultural pathogen to vaccine vector. Pathogens 10:1092. doi: 10.3390/pathogens10091092 34578125 PMC8470541

[ref19] Mire C. E. Geisbert J. B. (2013). Vesicular stomatitis virus-based vaccines protect nonhuman primates against Bundibugyo ebolavirus. PLoS Negl. Trop. Dis. 7:e2600. doi: 10.1371/journal.pntd.0002600, PMID: 24367715 PMC3868506

[ref20] Mishra A. R. Byrareddy S. N. (2020). IFN-I independent antiviral immune response to vesicular stomatitis virus challenge in mouse brain. Vaccines 8:326. doi: 10.3390/vaccines8020326 32575459 PMC7350232

[ref21] Myscofski D. M. Dutton E. K. (2001). Cleavage and purification of intein fusion proteins using the *Streptococcus gordonii* spex system. Prep. Biochem. Biotechnol. 31, 275–290. doi: 10.1081/PB-100104909, PMID: 11513092

[ref22] Peck S. H. Chen I. (2011). Directed evolution of a small-molecule-triggered intein with improved splicing properties in mammalian cells. Chem. Biol. 18, 619–630. doi: 10.1016/j.chembiol.2011.02.014, PMID: 21609843 PMC3124510

[ref23] Perler F. B. Davis E. O. (1994). Protein splicing elements: inteins and exteins--a definition of terms and recommended nomenclature. Nucleic Acids Res. 22, 1125–1127. doi: 10.1093/nar/22.7.1125, PMID: 8165123 PMC523631

[ref24] Rainsford E. W. Harouaka D. (2010). Importance of hydrogen bond contacts between the N protein and RNA genome of vesicular stomatitis virus in encapsidation and RNA synthesis. J. Virol. 84, 1741–1751. doi: 10.1128/JVI.01803-09, PMID: 20007268 PMC2812390

[ref25] Reed L. J. Muench H. (1938). A simple method of estimating fifty per cent endpoints. Am. J. Epidemiol. 27, 493–497. doi: 10.1093/oxfordjournals.aje.a118408

[ref26] Rodriguez L. L. Pauszek S. J. (2002). Full-length genome analysis of natural isolates of vesicular stomatitis virus (Indiana 1 serotype) from North, Central and South America. J. Gen. Virol. 83, 2475–2483. doi: 10.1099/0022-1317-83-10-2475, PMID: 12237430

[ref27] Romero-Casanas A. Gordo V. (2020). Protein splicing: From the foundations to the development of biotechnological applications. Methods Mol. Biol. 2133, 15–29. doi: 10.1007/978-1-0716-0434-2_2 32144661

[ref28] Schnell M. J. Buonocore L. (1996). The minimal conserved transcription stop-start signal promotes stable expression of a foreign gene in vesicular stomatitis virus. J. Virol. 70, 2318–2323. doi: 10.1128/jvi.70.4.2318-2323.1996, PMID: 8642658 PMC190073

[ref29] Shah N. H. Muir T. W. (2014). Inteins: Nature's gift to protein chemists. Chem. Sci. 5, 446–461. doi: 10.1039/C3SC52951G, PMID: 24634716 PMC3949740

[ref30] Si L. Xu H. (2016). Generation of influenza A viruses as live but replication-incompetent virus vaccines. Science 354, 1170–1173. doi: 10.1126/science.aah5869, PMID: 27934767

[ref31] Steinhauer D. A. Domingo E. (1992). Lack of evidence for proofreading mechanisms associated with an RNA virus polymerase. Gene 122, 281–288. doi: 10.1016/0378-1119(92)90216-C, PMID: 1336756

[ref32] Tatsis N. Ertl H. C. (2004). Adenoviruses as vaccine vectors. Mol. Ther. 10, 616–629. doi: 10.1016/j.ymthe.2004.07.013, PMID: 15451446 PMC7106330

[ref33] Walker P. J. Firth C. (2015). Evolution of genome size and complexity in the rhabdoviridae. PLoS Pathog. 11:e1004664. doi: 10.1371/journal.ppat.1004664, PMID: 25679389 PMC4334499

[ref34] Whelan S. P. Ball L. A. (1995). Efficient recovery of infectious vesicular stomatitis virus entirely from cDNA clones. Proc. Natl. Acad. Sci. U. S. A. 92, 8388–8392. doi: 10.1073/pnas.92.18.8388, PMID: 7667300 PMC41162

[ref35] Yahalom-Ronen Y. Tamir H. (2020). A single dose of recombinant VSV-G-spike vaccine provides protection against SARS-CoV-2 challenge. Nat. Commun. 11:6402. doi: 10.1038/s41467-020-20228-7, PMID: 33328475 PMC7745033

[ref36] Yuen C. M. Rodda S. J. (2006). Control of transcription factor activity and osteoblast differentiation in mammalian cells using an evolved small-molecule-dependent intein. J. Am. Chem. Soc. 128, 8939–8946. doi: 10.1021/ja062980e, PMID: 16819890 PMC2519127

[ref37] Zhang Z. Zhao Z. (2022). Comparative immunogenicity analysis of intradermal versus intramuscular immunization with a recombinant human adenovirus type 5 vaccine against Ebola virus. Front. Immunol. 13:963049. doi: 10.3389/fimmu.2022.963049, PMID: 36119119 PMC9472118

