# Supplementary material for: Regulated control of virus replication by 4-hydroxytamoxifen-induced splicing
Source: Front Microbiol. 2023 Mar 13;14:1112580. doi: 10.3389/fmicb.2023.1112580 (PMC10040539; doi:10.3389/fmicb.2023.1112580)
Supplement: Supplementary file 1 [file Data_Sheet_1.docx]

Supplementary Material

Regulated control of virus replication by 4-hydroxytamoxifen-induced splicing

Zhenghao Zhao^1^，Busen Wang^1^, Shipo Wu^1^, Zhe Zhang^1^, Yi Chen^1^, Jinlong Zhang^1^, Yudong Wang^1^，Danni Zhu^1,2^，Yao Li^1^，Jinghan Xu^1^，Lihua Hou^1^*, Wei Chen^1^*

^1^ Beijing Institute of Biotechnology, No. 20 Dongdajie Street, Fengtai District, Beijing 100071, China.

^2^ Qingdao Special Servicemen Recuperation Center of PLA Navy, Qingdao 266071, Shandong, China

*Correspondence to: [houlihua@sina.com](mailto:houlihua@sina.com) (L.H), [cw0226@foxmail.com](mailto:cw0226@foxmail.com,) (W.C)

**This PDF file includes:**  Figures. S1 to S5

# Supplementary Figures

**
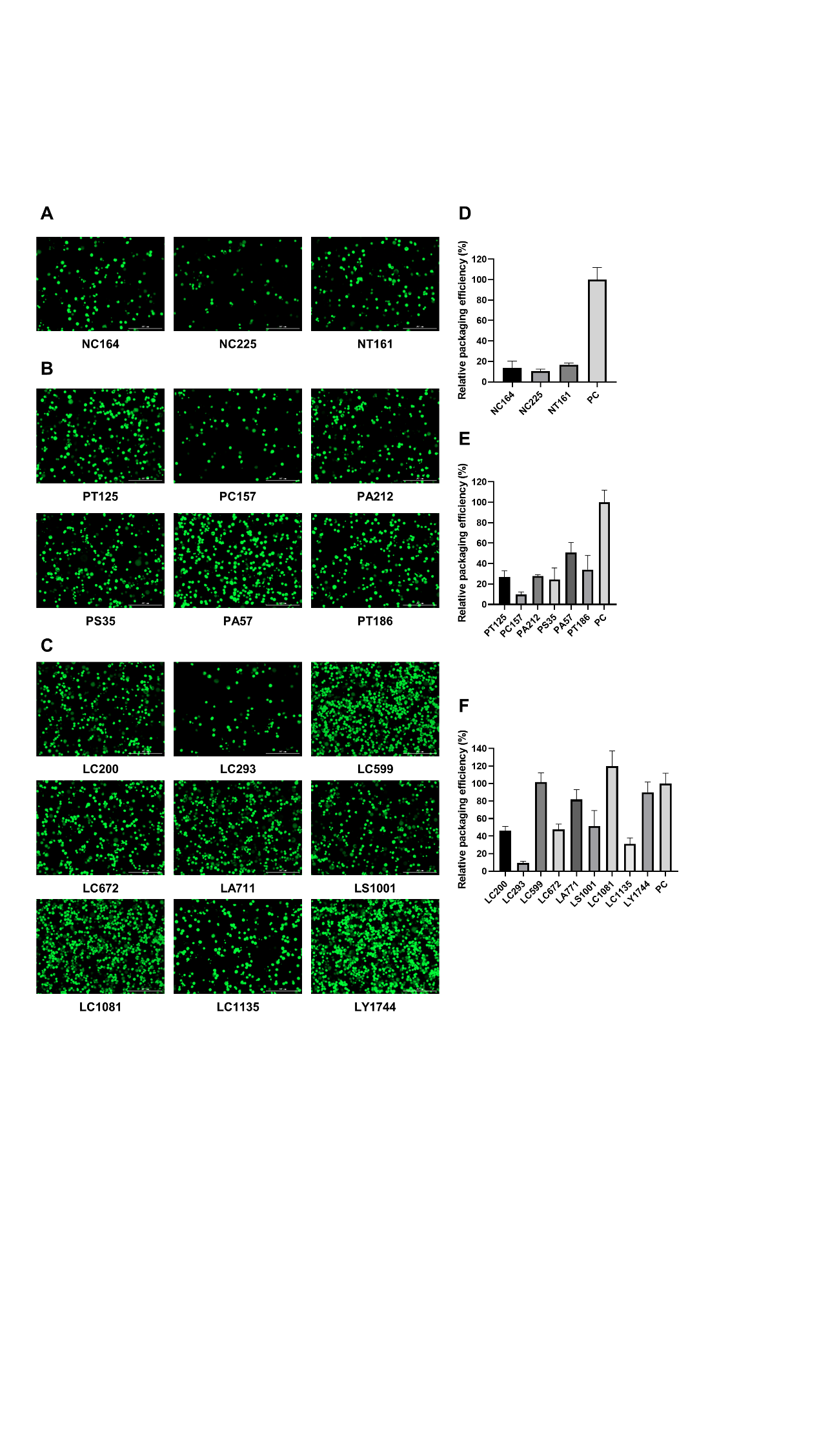
**

**Supplementary Figure 1. The packaging efficacy of recombinant VSV plasmid with different intein insertion-sites**

(A-C) Recombinant VSVs with intein inserting into N (A)/P (B)/L (C) protein infected the Vero E6 cells, and 3/6/9 sites on N/P/L protein could package into virus.

(D-E) Efficiency represents a normalization of the percentages of the FITC-positive cells compared to the VSV(dG)-GFP virus.

**
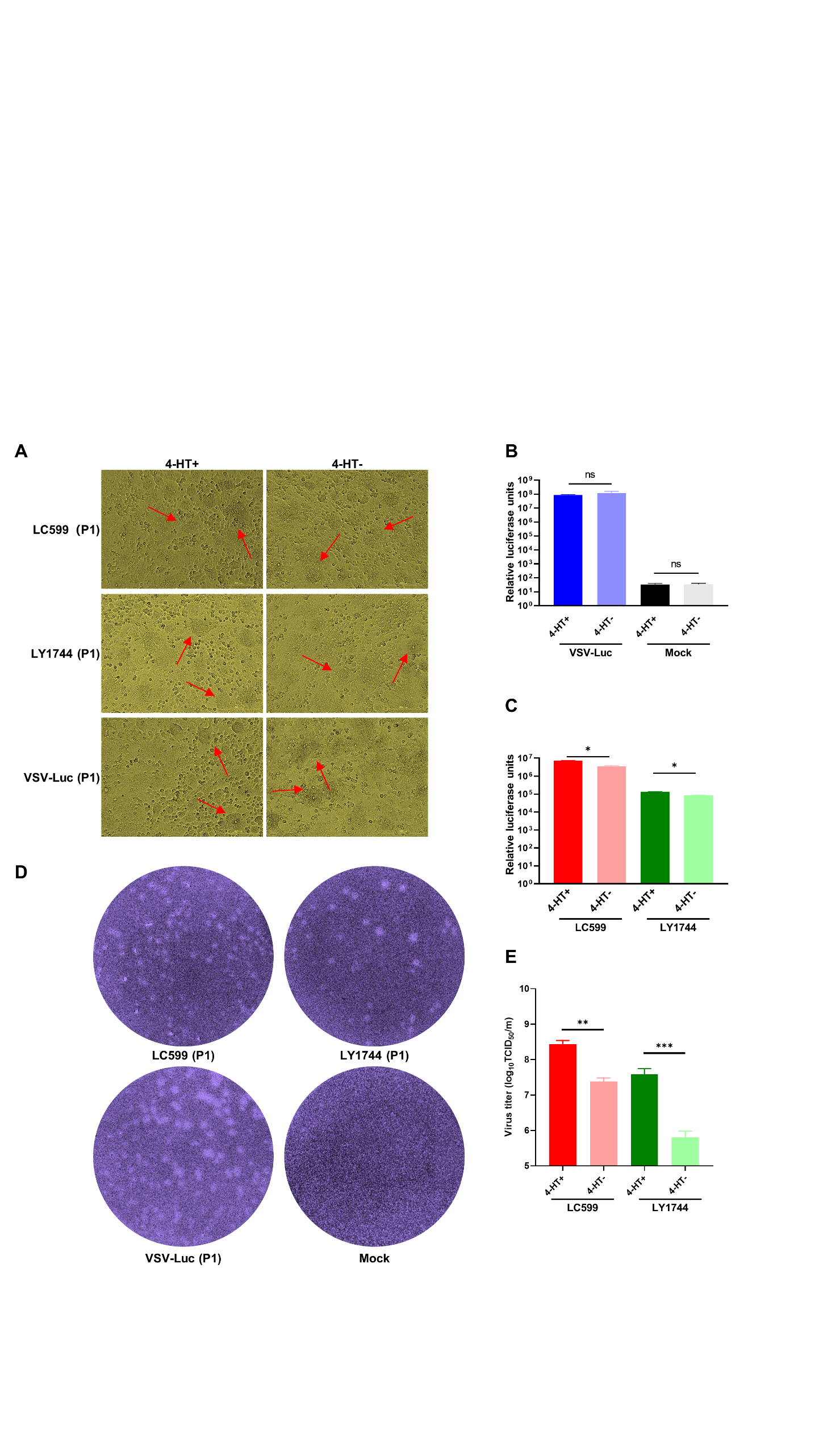
**

**Supplementary Figure 2.** Luciferase expression level of VSV-Luc (P2) and Mock (P2) with or without 4-HT

**
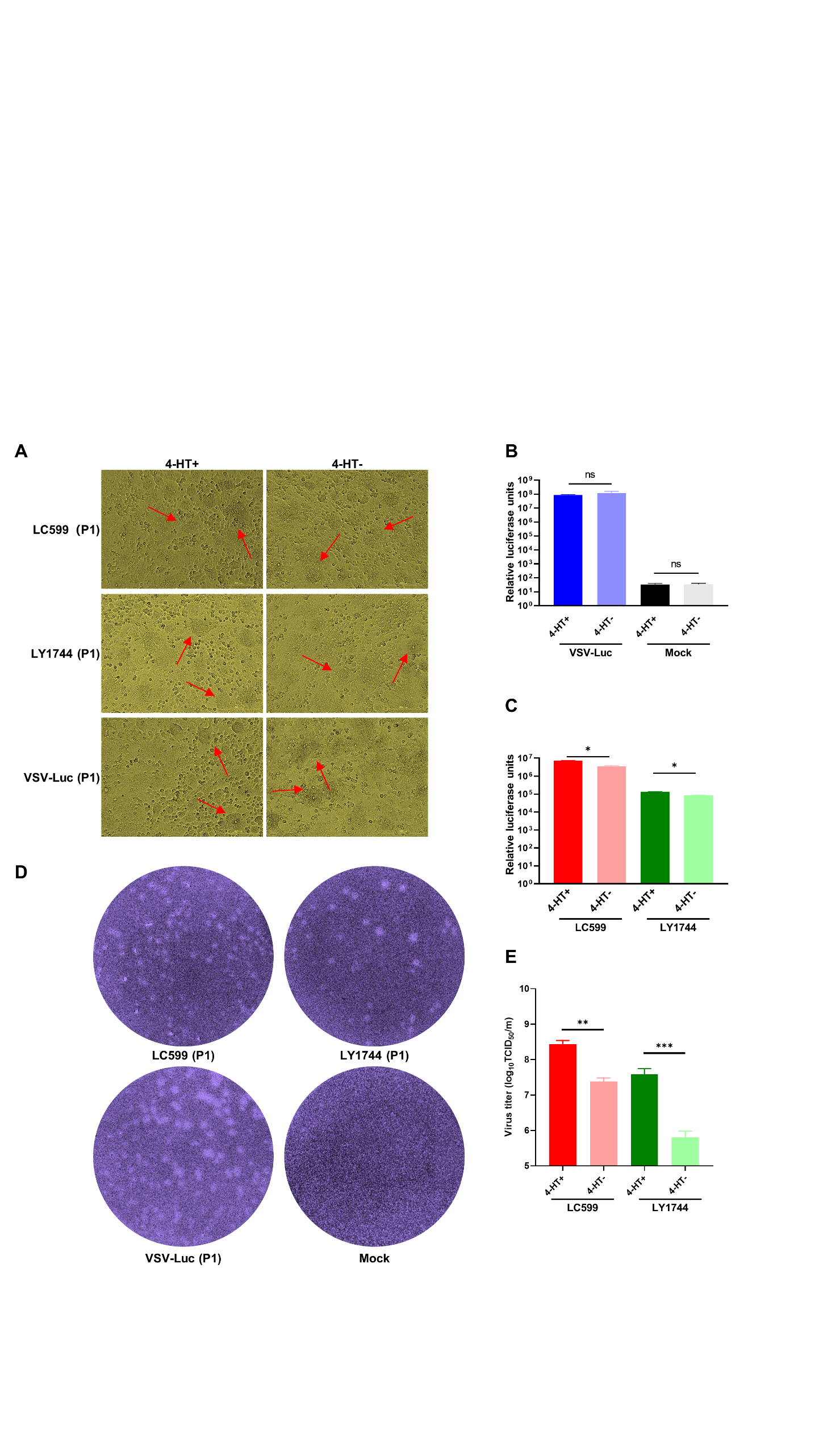
**

**Supplementary Figure 3** The titers of the progeny virus of the 5th passage of LC599/LY1744 cultured with or without 4-HT.

**
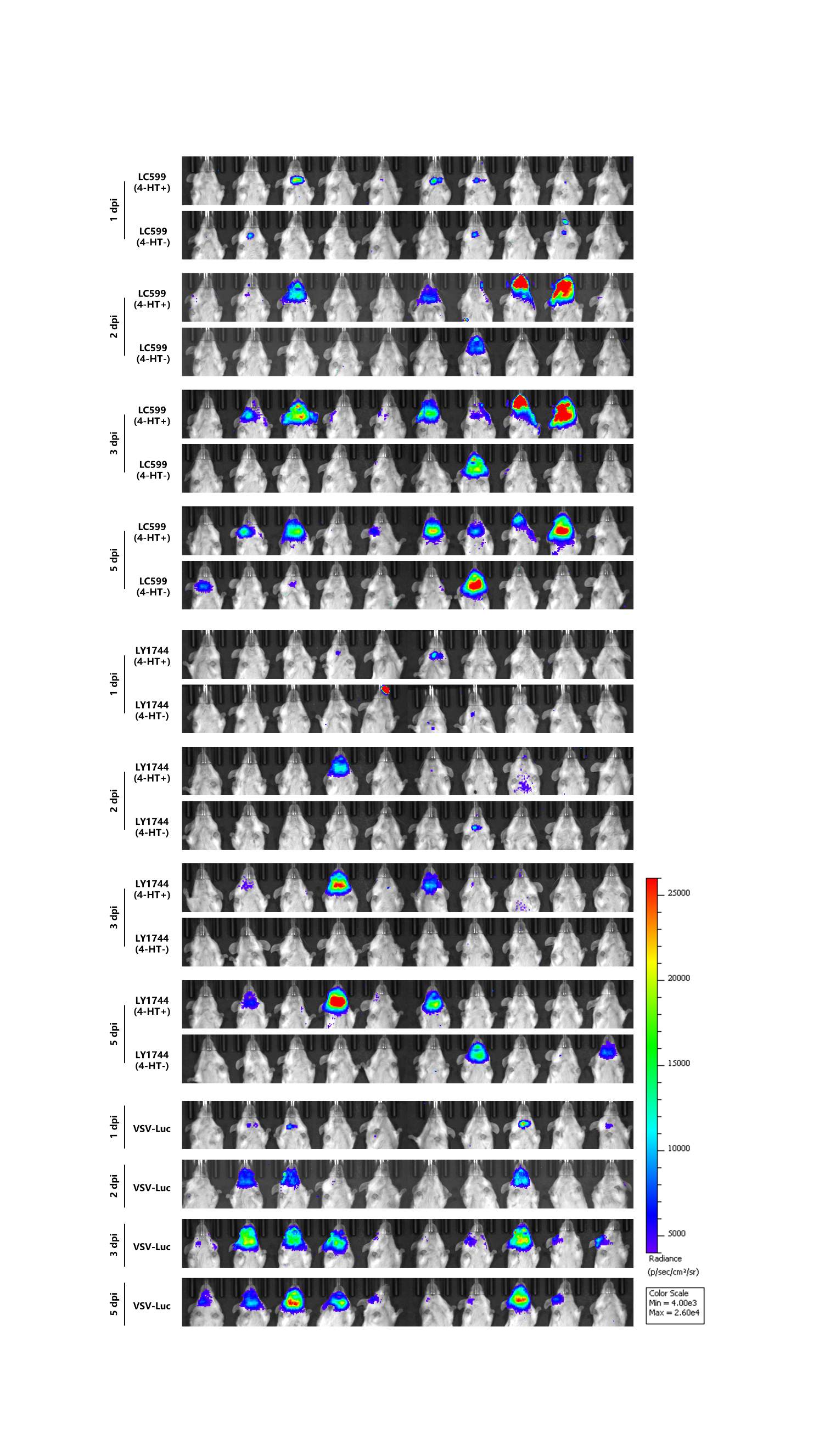
**

**Supplementary Figure 4.** Luminescence imaging in challenged mice on day 1，2，3 and 5. Luminescence intensity (photons/sec/cm2/sr) was represented in false colors.


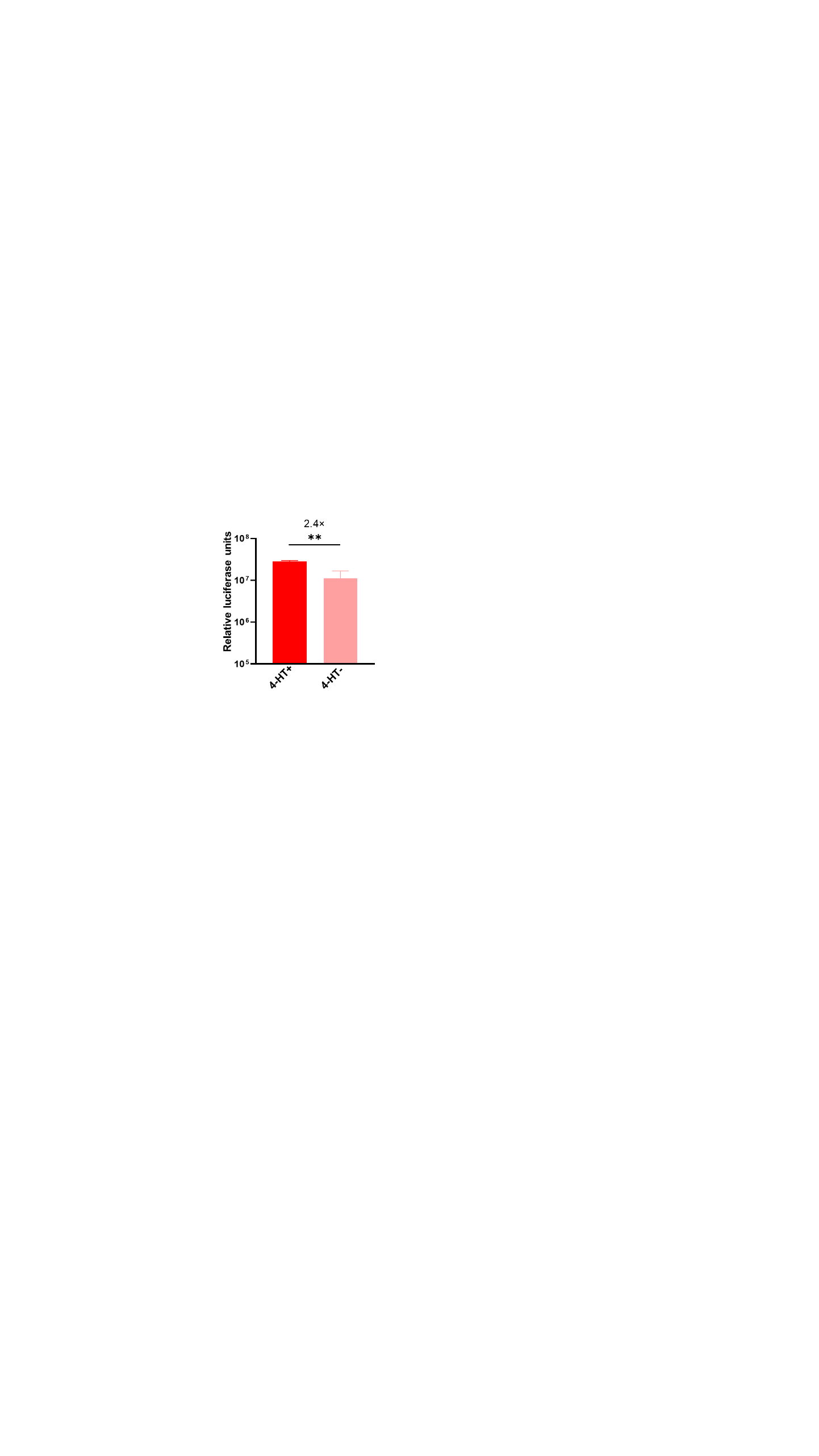


**Supplementary Figure 5.** The 4-HT regulation capacity of the virus extracted from the brain tissue of the seventh mouse in LC599 (4-HT^-^) group.

**The establish of BHK-T7 cell line**

The upstream primer 5 '-aacttaagcttgccgccaccatgaacacgattaacatcgc-3' and downstream primer 5 '-ctgcagaattcttacgcgaacgcgaagtcc- 3' were used to amplify the T7 RNAP sequence from genomic DNA of BL21. The PCR product was double digested with EcoRI and HindIII followed by ligation with a previously double-digested vector pcDNA3.1. BHK-21 cells were seeded in six-well plates and cultured until reached 90% confluence. The cells were transfected with 4 μg pcDNA3.1-T7 and passaged after 24 hours, then cultured and expanded with 400 μg/ml G418 for 2 weeks to obtain BHK-T7 cell pool. After that, single cell clones were selected and used for virus package.

**The construction of recombinant VSV plasmids and support plasmids**

The design of the sequence refers to the literature reported before ^[1,2]^. The sequence of VSV genome was obtained from NCBI (NCBI_ 001560.1), and the sequence of the HDV ribozyme and T7 terminator was added to the 3’ end of the VSV genome, and restriction sites XhoI and SbfI was added in the 5’ and 3’ of the gene of G protein, respectively. The total length of the sequence is 11347 bp, and it was subdivided into three fragments: 1-4006 (fragment 1), 4007-7626 (fragment 2) and 7627-11347 (fragment 3). The three fragments were synthesized and cloned into pUC57 vector by General Biol (CN), respectively. The pBluescript SK (+) vector was linearized by inverse PCR amplification. The three PCR fragments were amplified by PCR with a 20-25 bp homologous sequence added to each end of the fragments, then inserted into the linearized vector by homologous recombination (NEBuilder® HiFi DNA Assembly Master Mix) to construct the full-length VSV plasmid (pVSV).

VSV(d)G-GFP was constructed by homologous recombination of the PCR fragment of GFP, which had 23/24 bp homologous sequences to 5’/3 end of the fragments, and the double-digested vector (SbfI/XhoI) pVSV.

The pVSV was linearised by SbfI restriction enzyme digestion.The link gene 5’-gctagtctaa...tcgatctgttt-3’ between G and M and the luciferase gene were amplified by PCR and the 20-25 bp homologous sequences were added to each end of the fragments.The two PCR fragments were inserted into the linearized vector pVSV by homologous recombination to construct the VSV-Luc.

The fragments of N, P, G, L were amplified from the VSV genome with a T7 terminator fusing to the 3’ end and then the fragments were cloned into pBluescript SK (+) vector to construct pBlu-N, pBlu-P, pBlu-G and pBlu-L.

1. Lawson, N.D.; Stillman, E.A. Recombinant vesicular stomatitis viruses from DNA. *Proc Natl Acad Sci U S A* **1995**, *92*, 4477-4481.

2. Stillman, E.A.; Rose, J.K. Replication and amplification of novel vesicular stomatitis virus minigenomes encoding viral structural proteins. *J Virol* **1995**, *69*, 2946-2953.
